# Development of a population suppression strain of the human malaria vector mosquito, *Anopheles stephensi*

**DOI:** 10.1186/1475-2875-12-142

**Published:** 2013-04-26

**Authors:** Osvaldo Marinotti, Nijole Jasinskiene, Aniko Fazekas, Sarah Scaife, Guoliang Fu, Stefanie T Mattingly, Karissa Chow, David M Brown, Luke Alphey, Anthony A James

**Affiliations:** 1 Department of Molecular Biology & Biochemistry, University of California, Irvine, CA, 92697-3900, USA; 2 Oxitec Ltd, 71 Milton Park, Abingdon, Oxfordshire, OX14 4RX, UK; 3 Departments of Microbiology & Molecular Genetics and Molecular Biology & Biochemistry, 3205 McGaugh Hall, University of California, Irvine, CA, 92697-3900, USA; 4 Department of Zoology, University of Oxford, South Parks Road, Oxford OX1 3PS, UK

**Keywords:** *Anopheles stephensi*, Transgenic mosquitoes, Malaria

## Abstract

**Background:**

Transgenic mosquito strains are being developed to contribute to the control of dengue and malaria transmission. One approach uses genetic manipulation to confer conditional, female-specific dominant lethality phenotypes. Engineering of a female-specific flightless phenotype provides a sexing mechanism essential for male-only mosquito, release approaches that result in population suppression of target vector species.

**Methods:**

An approach that uses a female-specific gene promoter and antibiotic-repressible lethal factor to produce a sex-specific flightless phenotype was adapted to the human malaria vector, *Anopheles stephensi*. Transposon- and site-specific recombination-mediated technologies were used to generate a number of transgenic *An. stephensi* lines that when combined through mating produced the phenotype of flight-inhibited females and flight-capable males.

**Results:**

The data shown here demonstrate the successful engineering of a female-specific flightless phenotype in a malaria vector. The flightless phenotype was repressible by the addition of tetracycline to the larval diet. This conditional phenotype allows the rearing of the strains under routine laboratory conditions. The minimal level of tetracycline that rescues the flightless phenotype is higher than that found as an environmental contaminant in circumstances where there is intensive use of antibiotics.

**Conclusions:**

These studies support the further development of flightless female technology for applications in malaria control programmes that target the vectors.

## Background

Methods are being developed to control transmission of vector-borne diseases based on transgenic mosquitoes carrying a conditional dominant lethal gene (release of insects carrying a dominant lethal [RIDL]) [1-5]. The principal dengue virus vector, *Aedes aegypti*, was engineered in one RIDL-based approach to carry a transgene conferring a repressible female-specific flightless phenotype (fsRIDL) [2]. The sex-specificity of the phenotype results from the regulatory DNA of the *Ae. aegypti* gene, *Ae. aegypti Actin-4*[2,6], that encodes a female-specific flight muscle protein. These strains are showing promising results in meeting the goal of new tools for dengue vector population suppression although further refinements are necessary [7,8]. The technology also was shown to work in a related species, *Aedes albopictus*[4]. The technological advances achieved with *Aedes* species are potentially applicable to anophelines that transmit malaria parasites.

Features that contribute to the suitability of an fsRIDL-based, population-suppression tool for controlling malaria transmission include that the vector species is the major one responsible for malaria transmission in a defined geographical region and that controlling transmission will have a meaningful impact on local disease incidence and prevalence. In addition, the efficacy of such tools would be supplemented if the proposed species were endemic to a country that had in place robust malaria control programmes with which to interact and the target areas were of a reasonable scale. Geographic and ecological islands represented by confined urban and semi-urban areas would be ideal for evaluating the impact of the population suppression strain on the target species. Malaria transmission by *Anopheles stephensi* in urban and peri-urban settings in the Indian subcontinent appears to meet these criteria [9-11]. Malaria is endemic to the Indian subcontinent where 70–100 million cases per year are reported [12]. Successful application of molecular-genetic approaches to controlling malaria transmission by *An. stephensi* is expected to have a major effect on the disease burden of one of the most populous places in the world. *Anopheles stephensi* is recognized as having two forms that may be isolated reproductively and a third intermediate form [13,14]. The type-form is found principally in urban areas where it is responsible for the majority, if not all, of malaria transmission that occurs in cities. It breeds in peridomestic water containers and is highly anthropophilic. Distribution and feeding habits create functional “genetic islands” defined by breeding sites and hosts. A rural form, ‘*mysorensis’*, is found in the arid countryside and is not a major vector of malaria. The role of the intermediate form in transmission is unclear. Therefore, the urban mosquitoes are good targets for an fsRIDL-based, population-suppression tool.

The data presented here demonstrate that it is possible to engineer *An. stephensi* with a repressible female-specific flightless phenotype based on the use of the female-specific flight muscle promoter. These results support the prospect of applying fsRIDL in the fight against malaria on the Indian subcontinent.

## Methods

### Mosquito rearing

A colony of *An. stephensi* (gift of M Jacobs-Lorena, Johns Hopkins University) bred at the UCI insectary for longer than five years was used in the experiments. Insects were maintained at 27°C with 77% humidity and 12 hr day/night, 30 min dusk/dawn lighting cycle. Larvae were fed a diet of powdered fish food (Tetramin) mixed with yeast. Adults were provided *ad libitum* with a 0.3M sucrose solution in water and anesthetized mice were used for blood feeding when required. The blood-feeding protocol was approved by the Institutional Animal Care and Use Committee of the University of California, Irvine (NIH Animal Welfare Assurance number: A3416.01; approved February 20, 2008), Protocol Number: 1998–1411 (approved May 17, 2012). Transgenic and control non-transgenic (wild-type) mosquitoes were reared using the same standardized insectary procedures, unless otherwise indicated.

### Identification of the *Aedes aegypti Actin-4 gene* orthologue in *Anopheles stephensi*

Primary nucleotide sequences of the *Ae. aegypti Actin-4* (*AeAct-4*, AY531222) gene were used to search Vectorbase [15] for an orthologue in the genome and transcriptome of *An. stephensi*. The lowest e-value and highest score were assigned to ASTM009772. The reciprocal best BLAST hit strategy supports the 1:1 orthology of *AeAct-4* and ASTM009772. ASTM009772 is designated hereafter as *AsAct-4*.

Total RNA samples extracted with TRIZOL from larvae (mixed sexes), and separately from males and females, pupae and adults were treated with RNAse-free DNAse and served as templates for Reverse Transcriptase-PCRs, using a gene-specific pair of primers complementary to sequences located in the first and second exons of ASTM009772 (AsActin4 exon1for 5′-CACATAGTTGTTAGCTGGGAGAGC-3′, and AsActin4 5RACE 5′-CAGATCCATACGCAGGATAGCATG-3′).

The transcription start-site and intron location of *AsAct-4* was identified by 5′RACE and RT-PCR techniques. 5′RACE was performed using the Ambion FirstChoice RLM-RACE kit using the gene-specific primer AsActin4-5RACE. Comparison of cDNA (5′RACE and RT-PCR products) and *An. stephensi* genomic DNA, scaffold 01062 [15] sequences revealed one intron in the 5′UTR.

### Mosquito transgenesis, embryo microinjection and Southern-blot hybridization analyses

Microinjection of the pBac-IE1-DsRed2-AeAct4-tTA driver construct (OX3545) [2] with the *piggyBac* helper plasmid [16] in *An. stephensi* embryos was performed as described previously [17]. OX3545 contains the AeAct-4 promoter and first intron cloned at the 5′ end of tTAV linked to a SV40 3′ UTR, as described previously [2]. This gene cassette was inserted into a *piggyBac* vector containing the Hr5-IE1 promoter driving a gene encoding the fluorescent protein DsRed, to facilitate identification of transgenic mosquitoes. The helper plasmid phsp-pBac [16] has DNA sequences that drive constitutive expression of functional *piggyBac* transposase that catalyzes random insertions of the driver construct into the mosquito genome.

Each G_0_ male was mated with fifteen virgin females and groups of ten G_0_ females were mated with five males. The G_1_ progeny and the subsequent generations were screened at the larval stage with fluorescence microscopy for the expression of the DsRed marker gene. Transgenic lines were maintained as hemizygous by out-crossing transgenic individuals at every generation with wild-type mosquitoes. Southern blotting and hybridization techniques were used to detect transgene integration and copy number. Genomic DNA extracted from groups of six transgenic or wild-type control females was fractionated by agarose gel electrophoresis and transferred to Zeta-probe GT Genomic Tested Blotting Membranes (Bio-Rad, Hercules, CA) as described previously [18] and using standard protocols [19]. The probe used to identify transgene integrations consisted of the DsRed2 ORF and was amplified from the OX3545 plasmid using the primers 5′-GCAGCTGATCACGTACGCTC-3′ and 5′-GTGCGCTCGTACTGCTCCAC-3′ and labelled with ^32^P using the Megaprime DNA labelling system (Amersham).

The *AsAct-4* promoter and 5′UTR were used to make the driver construct, pBac-IE1-DsRed2-AsAct4-tTA (AsOX3545). A DNA fragment including 1014 base pairs (bp) of the 5′-end gene sequence, the first exon (96 bp), and the complete intron (271 bp) was amplified with the primers AsPforEcoRV (5′-GATATCCGAGTAGTGGGTGCTACTGTAGCG-3′) and AsPrevSpeI (5′-ACTAGTGCCGAGTGTTATGGATTGTCCTGCAGG-3′). The amplified DNA was digested with *Eco*RV and *Spe*I restriction endonucleases and the resulting fragment cloned into OX3545 digested previously with *Nru*I and *Spe*I. Microinjection of AsOX3545 in *An. stephensi* embryos and analyses of the resulting transgenic lines were performed as described above.

The effector construct attB-3×P3-DsRed2-tetO-Nipp1Dm (OX3547) [2] was injected into embryos of the docking-site strain, *attP*43 [20] together with *in vitro-*transcribed *φC31* integrase mRNA [21]. The *φC31* integrase catalyses recombination between the *attB* site in the plasmid and the *attP* site in the genome of the docking site strain, promoting site-specific integration of transgenes in the target genome. OX3547 contains the regulatory sequence of a tetracycline responsive element (tRE), comprising multiple copies of the tetO sequence, to which tTA binds, and a basal promoter driving expression of the effector molecule, Nuclear Inhibitor of PP1 (Nipp1Dm). Nipp1Dm overexpression induces phenotypes that include lethality, abnormal mitotic figures and defects in muscle development [2,22,23]. tTA cannot bind to tetO in the presence of tetracycline and expression of Nipp1Dm remains uninduced.

The survivors of OX3547 injections were grouped into female and male pools and out-crossed with wild-type, non-transgenic mosquitoes of the opposite sex. The offspring were screened as larvae with fluorescence microscopy for the expression of the fluorescent DsRed marker. Genomic DNA extracted from groups of six females was fractionated by agarose gel electrophoresis and transferred to Zeta-probe GT Genomic Tested Blotting Membranes (Bio-Rad, Hercules, CA). Southern blots to evidence transgene integration were performed as described above and used the same ^32^P-labelled DsRed2 ORF probe.

### Generation of conditional flightless mosquitoes and tetracycline rescue of the phenotype

Conditional flightless female mosquitoes were generated by crossing males or females hemizygous for the driver constructs with those of the opposite sex hemizygous for the effector construct. Progeny were screened as larvae with fluorescence microscopy for the expression of the marker genes. The DsRed2-encoding marker gene is the same in the driver and effector lines, however the driver lines express red fluorescent protein in a scattered pattern throughout the body under the control of the IE1 promoter while DsRed2 expression in the effector lines is driven by the eye-specific promoter, 3×P3. Progeny of crosses of the driver and effector lines presenting both the IE1 and the 3×P3 driven fluorescence patterns were selected for further analyses. The double-hemizygous individuals were raised to adults and the survival of males and females as well as frequency of adult flightless phenotype scored. Adult females were considered flightless when they could not elevate and remain aloft after the mosquito cages were agitated. Flightless females tend to walk, may be able to hop but do not fly for even short distances. Alternatively, double-hemizygous progeny were raised in the presence of tetracycline added to the water during larval development. Adults were offered a 0.3M sucrose solution supplemented with tetracycline (the same concentration used for rearing larvae) and allowed to feed *ad libitum*. The survival of tetracycline-treated males and females, and frequencies of marker gene and adult flightless phenotypes were scored.

## Results

### Transgene constructions

**Figure 1:**
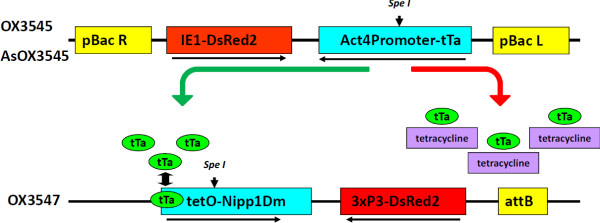
**Schematic representations of the transformation constructs OX3545, AsOX3545 and OX3547, and the mechanism of action of the double transgene-based female-specific RIDL.** OX3545 and OX3547 were previously described in detail [2]. AsOX3545 is a similar to OX3545, with the *Anopheles stephensi Actin-4* regulatory sequences replacing the *Aedes aegypti Actin-4* sequence. *Actin 4* promoters (*Act4P*) drive the expression of the tetracycline-repressible transactivator protein (tTa) in the flight muscles of adult female mosquitoes. tTA binds tetO in the absence of tetracycline, driving expression of the effector molecule, Nuclear Inhibitor of PP1 (Nipp1Dm) [22,23], leading to disruption of normal cell functions. Tetracycline prevents tTa binding to tetO abolishing expression of Nipp1Dm. The positions of the *Spe*I recognition and cleavage sites in OX3545, AsOX3545 and OX3547 are indicated by vertically oriented arrows. Horizontally oriented arrows indicate the direction of transcription for each component of the constructs. Abbreviations: pBac R and L, *piggyBac* right and left inverted repeats, respectively; *attB, φC31* recombination site; DsRed2, red fluorescent protein open reading frame; IE1, baculovirus immediate-early gene promoter driving DsRed2 expressed throughout the body; 3×P3, artificial promoter consisting of a multimer of the binding site (P3), driving high levels of expression in the eye.

Two existing transgene constructs, previously used for genetic transformation of *Ae. aegypti*, and a third, novel construct were used in the experiments (Figure 1). Replacement of the *AeAct-4* gene 5-end sequence (putative promoter and first intron) with the orthologous DNA from *AsAct-4* originated the novel driver construct (AsOX3545). Putative orthology of the *Ae. aegypti AeAct-4* and *An. stephensi* ASTM009772 genes was assigned by sequencing (Additional file 1: Figure S1) and reciprocal Blast analysis. Blastp using the predicted *AeAct-4* protein sequence as query identified ASTM009772-PA as the best hit within the *An. stephensi* proteome (E-value = 0.0, Score = 2010, Identity = 99.2%). The reciprocal blast, using ASTM009772-PA as query identified *AeAct-4* as the best hit within the *Ae. aegypti* proteome (E-value = 0.0, Score = 2010, Identity = 99.2%). The orthology assignment is supported further by gene expression analyses showing that in *An. stephensi* ASTM009772-RA transcripts are present in fourth instar larvae (mixed sexes) and female pupae, and absent in male pupae and adults, a pattern similar to that of *AeAct-4*[2,6].

### Generation of transgenic driver lines

**Figure 2:**
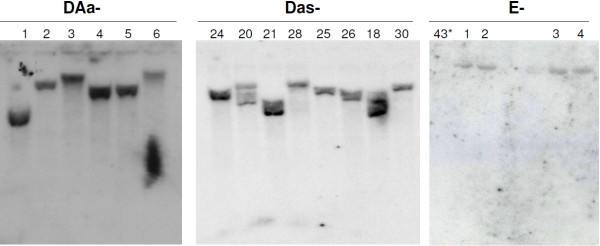
**Southern blot detection of transgenes integrated into the genomes of *Anopheles stephensi *driver and effector transgenic lines.** Genomic DNA extracted from a pool of mosquitoes from each line was digested with *SpeI*, fractionated by agarose gel electrophoresis, transferred to a Zeta-probe GT membrane and hybridized to a ^32^P-labelled DsRed2 open-reading frame probe. DAa, DAs and E and the numbers on top of the figures indicate the individual transgenic lines originated from injection of the OX3545 and AsOX3534 driver and OX3547 effector constructs, respectively.

A total of 1,423 *An. stephensi* embryos injected with the OX3545 driver construct and *piggyBac* helper plasmid resulted in 447 (31%) individuals surviving and developing into adults. These G_0 _adults were back-crossed with the wild-type, non-transgenic recipient strain and 31 produced DsRed2 fluorescent G_1_ larvae for an estimated transformation efficiency of ~7%. All DsRed2 fluorescent progeny from 16 of the 31 outcrosses died during their pre-imago stages and hence adults were recovered from only 15 matings. These transgenic lines were designated DAa-*xx*, indicating that they are driver lines carrying a transgene with the *Ae. aegypti Actin-4* regulatory sequence. Eight of the G_1_ driver lines (DAa-1, 2, 3, 4, 5, 6, 7 and 8) produced viable transgenic offspring when crossed with wild-type mosquitoes (Table 1). All driver lines derived from injections with the OX3545 construct exhibited some degree of lethality during metamorphosis of the G_2_ mosquitoes, with line DAa-1 showing only 8% survival. Survival in line DAa-1 increased to 24% by the fifth generation (G_5_) post injection (Additional file 2: Table S1). All driver lines produced a higher percentage of adult males in generations G_2 _through G_5 _than was observed in control lines. Southern blot analyses of genomic DNA isolated from G_5 _adult mosquitoes indicate distinct transgene integration events in each of the characterized lines (Figure 2). All lines had hybridization signal consistent with a single insertion of the driver construct in the *An. stephensi* genome.

**Table 1:** **Sex ratio and survival of driver transgenic lines carrying the *Aedes aegypti Act-4 *promoter (OX3545 construct)**

**G**_**0**_	**G**_**1**_	**G**_**2**_			
**Lines outcrossed with wild-type**	**Transgenic adults**	**Wild type larvae**	**Transgenic larvae**	Transgenic adults*	
				**Females**	**Males**
DAa-1 ♂	1 ♂	325	199	0	17
DAa-2 ♂	4 ♂	680	138	21	22
DAa-3 ♂	1 ♂	200	86	3	29
DAa-4 ♂	17 ♂	1140	659	31	134
DAa-5 ♂	4 ♂	90	45	3	11
DAa-6 ♀	1 ♂	70	44	22	9
DAa-7 ♀	1 ♂	100	45	9	15
DAa-8 ♀	1 ♂	90	30	10	17
DAa-9 ♂	1 ♂, 2 ♀	0	0	0	0
DAa-10 ♂	1 ♂	0	0	0	0
DAa-11 ♂	2 ♂	0	0	0	0
DAa-12 ♂	1 ♂	0	0	0	0
DAa-13 ♂	2 ♂	0	0	0	0
DAa-14 ♂	1 ♂	0	0	0	0
DAa-15 ♂	1 ♀	0	0	0	0

*A flightless phenotype was not observed in males or females. An expanded Table showing results of five generations is provided as Additional file 2: Table S1.

The driver construct containing the *An. stephensi Actin-4* regulatory DNA, AsOX3545, was injected in 995 embryos, resulting in 408 (41% survival) G_0_ adults. Twenty-one transgenic lines (~5% transformation efficiency), designated DAs-*xx*, were generated after out-crossing G_0_ adults with the wild-type strain (Table 2). For one line, DAs-16, all transgenic females were flightless. This phenotype was not repressed by rearing on tetracycline. Southern blot analyses revealed a variable number of transgene insertions, ranging from one (lines DAs-28 and DAs-30) to at least four in line DAs-20 (Figure 2).

**Table 2:** **Sex ratio, survival, and flightless phenotype of driver transgenic lines carrying the *Anopheles stephensi Act-4 *promoter (AsOX3545 construct)**

**G**_**0**_	**G**_**1**_	**G**_**2**_			
**Lines outcrossed with wild-type**	**Transgenic adults**	**Wild-type larvae**	**Transgenic larvae**	**Transgenic adults**	
				**Females**	**Males**
				**flightless/flying**	**flying**
DAs-16	1 ♂	120	119	33/0*	33
DAs-17	17 ♂	300	101	4/21	36
DAs-18	33 ♂	300	131	2/19	51
DAs-19	1 ♂	210	129	0/53	48
DAs-20	2 ♂	320	321	0/21	65
DAs-21	4 ♂	510	202	0/37	95
DAs-22	1 ♂	260	176	0/21	48
DAs-23	1 ♂	170	147	0/19	52
DAs-24	1 ♂	410	344	0/19	32
DAs-25	14 ♂	350	168	0/19	35
DAs-26	27 ♂	400	107	0/29	39
DAs-27	4 ♂	70	62	0/4	20
DAs-28	1 ♂	130	163	0/3	35
DAs-29	10 ♂	410	178	0/8	17
DAs-30	1 ♂	120	144	0/9	6
DAs-31	1 ♀	300	46	0/3	3
DAs-32	1 ♀	500	32	0/4	14
DAs-33	1 ♀	450	37	0/4	3
DAs-34	2 ♂	90	54	0/0	3
DAs-35	1 ♀	400	47	0/0	6
DAs-36	3 ♂	40	13	0/0	1

*The flightless female phenotype of line DAs-16 is not rescued by tetracycline.

### Generation of transgenic effector lines

The *An. stephensi* transgenic line *attP43* has two *φC31 attP* docking sites in its genome [20]. Five hundred line *attP*43 embryos injected with the attB-3×P3-DsRed2-tetO-Nipp1Dm effector construct (OX3547) produced 178 (36% survival) adult mosquitoes (Table 3). The surviving adults were reared as 11 male and eight female pools and out-crossed with wild-type mosquitoes. The progeny of four pools, designated E-1 to E-4, contained transgenic individuals detected by fluorescence microscopy. The sex ratio of the progeny is consistent with the inserted transgene being integrated into the X chromosome. Genomic DNA extracted from G_1_ transgenic mosquitoes analyzed with Southern blots revealed that the effector transgene was inserted into the same X-linked *φC31 attP* docking site in all four integration events (Figure 2). Adults of the four transgenic lines were combined in a single population and named E-1.

**Table 3:** Sex-ratio and survival of effector transgenic lines carrying the OX3547 construct

**G**_**0**_	**G**_**1**_	**G**_**2**_			
**Effector lines outcrossed with wild-type**	**Transgenic adults**	**Outcross**	**Transgenic larvae**	**Transgenic adults**	
				**Females**	**Males**
E-1 (♂)	0 ♂, 28 ♀	G1 ♀ x WT* ♂	57	29	26
E-2 (♂)	0 ♂, 42 ♀	G1 ♀ x WT ♂	128	59	62
E-3 (♀)	6 ♂, 2 ♀	G1 ♂ x WT ♀	81	76	0
E-4 (♀)	25 ♂, 26 ♀	G1 ♂ x WT ♀	32	27	0

*WT, wild-type.

### Crossings of DAa driver and E-1 effector lines

Seven DAa driver lines (DAa1-7) were crossed with the effector line E-1 and the flight capabilities and sex ratio of their progeny analysed (Table 4). Larvae hemizygous for both driver and effector transgenes were identified by the tissue-specific expression patterns of DsRed2 fluorescence consistent with those produced by the 3×P3 and the IE1 promoters (Additional file 3: Figure S2).

**Table 4:** Sex-ratio, survival, and flightless phenotypes in the F1 progeny of crosses between the effector line E-1 and driver lines engineered with either OX3545 (DAa-) or AsOX3545 (DAs-)

**♂ driver lines outcrossed with the ♀ effector line E-1**	**Larvae**		**Transgenic adults**			
	**Only one transgene**	**Effector and driver transgenes**	**Flying females**	**Flightless females**	**Flying males**	**Totals(% survival larvae-adult)**
DAa-1	775	144	0	1	62	63 (44%)
DAa-2	1150	307	7	0	34	41 (13%)
DAa-3	4340	880	9	172	345	517 (70%)
DAa-4	600	110	3	23	29	54 (49%)
DAa-5	900	226	47	0	96	143 (63%)
DAa-6	1830	371	140	0	143	283 (76%)
DAa-7	1200	239	41	6	74	121 (51%)
DAs-17	nd	465	80	79	177	336 (72%)
DAs-18	nd	89	11	3	23	37 (41%)
DAs-19	nd	185	58	20	62	140 (75%)
DAs-20	nd	152	11	0	52	63 (41%)
DAs-21*	nd	1545	0	161	483	644 (42%)
DAs-22	nd	1483	3	397	507 (1)**	907 (61%)
DAs-24	nd	93	23	4	28	55 (59%)
DAs-25*	nd	761	0	70	193	263 (35%)
DAs-26	nd	328	15	86	112	213 (65%)
DAs-28	nd	244	19	0	73	92 (37%)
DAs-29	nd	107	33	0	33	66 (61%)
DAs-30	nd	226	15	0	14	29 (12%)
DAs-34	nd	114	8	0	0	8 (0%)
DAs-36	nd	267	21	0	46	67 (25%)

*Lines with design-feature phenotypes.

**Crossing Das-22 and E-1 lines generated one flightless male (indicated in parenthesis).

Larvae carrying both the effector and the driver transgenes are expected to represent 25% of the progeny. ^*2*^ analyses of crosses between males from DAa lines and E-1 females resulted in significantly reduced proportions of double-hemizygous larvae relative to this expectation (*X*^*2*^ from 11.79 to 184.11; df = 1, and p < 0.001 for all crosses). These results support the interpretation that some load is associated with carrying the transgenes. The surviving double-hemizygous individuals were maintained in the absence of tetracycline and allowed to develop into adults. Crosses with driver line DAa-1 produced 97% adult male progeny, reflecting significant lethality in developing females. The single adult female recovered from this cross exhibited a flightless phenotype. In contrast, crosses with driver line DAa-6 males resulted in equal numbers of adults of both sexes and no flightless phenotypes were observed among the surviving females. In addition, the flightless phenotype was not observed in any female of crosses involving males from DAa-5 or DAa-2. Driver lines DAa-3 and DAa-4 crossed with E-1 produced results consistent with the design intent of the transgenes with 95% of the females exhibiting the flightless phenotype and all males capable of flight.

### Crossings of DAs driver and E-1 effector lines

Eight of 14 DAs driver lines produced progeny with a flightless-female phenotype following crossing with the effector line E-1 (Table 4). The majority of the female progeny of lines DAs-24 (15% flightless) and DAs-18 (21% flightless) was able to fly. Line DAs-17 produced similar numbers of flying and flightless females. Lines DAs-26 and DAs-22 produced mostly flightless females (85% and 99% flightless, respectively) while DAs-21 and DAs-25 crosses with E-1 resulted in 100% flightless females. The sex-ratios of the adult progeny resulting from DAs-21, DAs-25 and DAs-22 crosses with E-1 are abnormal (*X*^*2*^ from 13 to 160; df = 1, and p < 0.001 for all crosses) with more males than females indicating some degree of female lethality at the sub adult stages. No sex-associated lethality was observed when crossing lines DAs-24, DAs-26, DAs-17 and DAs-19 (*X*^*2*^ from 0.0 to 0.9; df = 1, and p > 0.2 for all crosses).

### Tetracycline suppression of the flightless phenotype

Tetracycline provided to double-hemizygous transgenic *An. stephensi* is expected to rescue the flightless-female phenotype by preventing tTA binding to tetO and therefore inhibiting expression of the effector molecule Nipp1Dm. Eggs (estivated embryos) were collected from E-1 females mated with DAa-3, DAs-21 or DAs-22 driver line males and allowed to develop either in the presence or absence of tetracycline (Tables 4 and 5). The presence of tetracycline results in adults of both sexes with the flying, wild-type phenotype. All adult males except one could fly independent of the presence of tetracycline. In contrast, 98% (731/743 combined for DAa-3, DAs-21 and DAs-22; Table 4) of the adult females reared in the absence of tetracycline were flightless. The majority, ≥ 67%, of the females treated with ≥ 1 μg/ml tetracycline were able to fly, while tetracycline concentrations of 100 and 10 ng/ml only rescued partially (48 and 24%, respectively) the flightless phenotype (Table 5).

**Table 5:** **Effects of tetracycline on survival and flightless phenotype of transgenic *Anopheles stephensi *carrying driver and effector transgenes**

**♂ driver lines outcrossed with ♀ effector line E-1**	**Tetracycline concentration**	**Larvae**		**Transgenic adults**			
		**Only one transgene**	**Effector + driver transgenes**	**Flying females**	**Flightless females**	**Flying males**	**Totals (% survival larvae-adult)**
DAa-3	10 μg/ml	2535	371	108	16	226	350 (94%)
DAa-3	5 μg/ml	930	269	37	3	91	131 (49%)
DAa-3	1 μg/ml	610	143	21	10	69	100 (70%)
DAa-3	100 ng/ml	750	162	15	16	61	92 (57%)
DAa-3	10 ng/ml	600	127	9	28	38	75 (59%)
DAs-21	10 μg/ml	nd	233	25	18	82	125 (54%)
DAs-22	10 μg/ml	nd	628	155	12	285 (1)*	453 (72%)

*One flightless male was observed among the progeny of driver DAs-22 ♂ x effector E-1 ♀ cross (indicated in parenthesis).

## Conclusions

The results shown here demonstrate that the genetics and resulting phenotypes of a female-specific RIDL strategy, previously developed for dengue vector mosquitoes [2,4] can be adapted to a vector of human malaria, *An. stephensi*. This demonstration supports efforts to optimize the efficacy of the gene constructs and to eventually perform cage and field trials to assess the usefulness of this approach as a malaria vector population suppression tool.

It is clear that further refinements of this system are required. Efficient scale-up would benefit from having to manage only a single line, so linkage of the driver and effector genes would ultimately be required. Furthermore, *piggyBac*-mediated transposition was used to insert randomly the driver constructs into the mosquito genome. The expectation was that position effects resulting from the activity state of the DNA surrounding each independent insertion site would influence in a variable manner the expression of the driver construct. This was evident in variable penetrance of the intended flightless phenotype and the sex-independent and -dependent lethality in some lines. Remarkably, Southern blot analyses showed that all of the recovered driver lines carrying the *AeAct-4* promoter and at least two of those carrying the *AsAct-4* promoter had single transgene insertions. This is unusual for *piggyBac* integration into *An. stephensi*[20,24] and supports the conclusion that the driver construct alone can be deleterious if present in high-copy number as well as being integrated at a chromosomal site that enhances its expression. Selection and characterization of favourable chromosomal docking sites, site-specific recombination and DNA fragments with insulator-like activity would assist the development of a strain with the optimized flightless phenotype and diminish or eliminate male fitness impact [20,24,25].

Scale-up in testing of novel genetic strains is needed to ensure that the engineered mosquitoes function as designed in the field. The *Ae. aegypti* fsRIDL line OX3604C had outstanding performance characteristics in small and large laboratory cage trials, but did not meet efficiency expectations derived from modelling when tested in large outdoor field cages [7,8]. It is important to note that the field-cage trials were limited to only one test of a single, independently-derived fsRIDL strain. Here again, strain optimization using alternative characterized genome insertion sites, DNA insulator sequences [24] and even redesigning the transgenes could produce a strain that can be used with the expectation of an epidemiological impact on pathogen transmission.

The translation of new technologies from the laboratory to the field requires that resulting products function in a safe and efficacious manner. Transgenic mosquito technologies bring with them a number of challenges that can be addressed in the design features of the final products and site-specific community engagement activities [26-28]. A specific concern about the conditional, dominant, lethal genes is whether environmental contamination by tetracycline could produce a hazard by rescuing the flightless phenotype in the released transgenic mosquitoes. The most plausible source of rescue would be tetracycline contaminating larval habitats. No reports exist of measurements of tetracycline in the urban breeding sites of *An. stephensi*. However, there is an extensive literature on the widespread use of antibiotics for therapeutic, prophylactic and growth promotion purposes in livestock production. Antibiotics, including tetracycline and its analogues, are transferred to soils and surface waters when manure from the animals is spread on farmland or held in wastewater ponds. Several studies have been conducted to measure the concentrations of antibiotics in wastewater, animal waste and soil from manure-treated farmland. In studied sites, concentrations of tetracycline, tetracycline analogues or tetracycline degradation products were found to be lower than 1 μg/ml. One of the highest numbers measured was 0.698 ng/ml tetracycline in farmland treated with pig-manure slurry [29]. Other locations, such as waste-water treatment facilities, where persistent tetracycline could come from various sources such as cities and rural areas, had a maximum tetracycline concentration of 0.004 μg/ml [30]. The highest detected concentration of chlortetracycline in the same study was estimated at 0.0012 μg/ml. These concentrations are atypical, though. Bodies of water such as rivers and streams where runoff from farms could potentially be problematic contained minimal amounts of tetracycline, with concentrations below the limit of detection (≤ 0.5 ng/ml) [31]. Reviews examining tetracycline levels measured in waste-water treatment plants in USA and Asia found concentrations in the range <0.00003-0.62 ng/ml [32,33]. These data indicate that environmental tetracycline (and tetracycline analogue) concentrations detected in the breeding sites in which *An. stephensi* larvae are most likely to be located are not sufficient to rescue the engineered mosquito flightless phenotype.

*Anopheles stephensi* is an important malaria vector in the Indian subcontinent. Therefore the management of *An. stephensi* populations is of prime importance to public health and welfare. Successful population suppression could result in local or regional vector elimination dependent on the scope and size of the effort. It is anticipated that this suppression would result in less parasite transmission and therefore reduced human morbidity and mortality. However, population suppression technologies would have to be sustained to prevent re-colonization of the treated areas following vector migration or natural- or human-aided dispersal. Sustainable transgenic technologies also are being developed and these may represent second-generation genetic tools for controlling parasite transmission [25]. In the interim, the data presented here support the conclusion that fsRIDL should be developed as a tool in integrated pest management practices to control malaria transmission in urban settings were *An. stephensi* is the major vector.

## Competing interests

Those authors affiliated to Oxitec Limited (SS, GF and LA) are employees of this company, which provided salary and other support for the research programme. Such employees may have shares or share options in Oxitec Ltd. Both Oxitec Ltd and Oxford University have one or more patents or patent applications related to the subject of this paper.

## Authors’ contributions

OM, NJ, LA, DMB, and AAJ were involved in the conceptualization, research design, data collection and preparation of the manuscript. AF, SS, GF, SM, and KC contributed to reagent development and data collection. All authors read and approved the final manuscript.

## Supplementary Material

Additional file 1: Figure S1 Primary nucleic acid sequence and schematic representation of the *Anopheles stephensi Actin-4* gene. A) Capitalized nucleotide sequences correspond to exons 1 and 2, 96 and 1155 nt in length, respectively, while lower case indicates the 1012 nt 5′-end flanking sequence and the 271 nt intron. Underlined sequences indicate the locations of primers described in the manuscript and the transcription (+1) and translation (ATG) start sites are indicated underlined and in bold. B) Exons 1 and 2 (red boxes), the 5′ flanking sequence (green box) and the intron (yellow box) of the *An. stephensi Actin-4* gene. Lines underneath indicate the positions of the 1400 nt long AsActin4 regulatory region used to generate AsOX3545, the 5′ RACE product that identified the transcription start-site and the RT-PCR product utilized to determine the gene expression profile. C) Gene amplification analysis of the expression profile of *AsAct-4*. The 536 nt amplicon is specific for the gene product. Template mRNA samples were derived from larvae (L), pupae (P) and adults (A). An amplification product specific to the 18s rRNA is the positive sample control. Click here for file

Additional file 2: Table S1 Sex ratio and survival of *Anopheles stephensi* driver transgenic lines (DAa-) carrying the construct OX3545. Click here for file

Additional file 3: Figure S2 *Anopheles stephensi* larvae hemizygous for driver and/or effector transgenes were identified by patterns of DsRed2 fluorescence consistent with those produced by the 3XP3 and the IE1 promoters. DAa-3 larvae display a scattered fluorescence pattern throughout the body driven by the IE1 promoter (A) while DsRed2 expression in the effector line E-1 is driven by the eye-specific promoter, 3XP3 (B). Larvae hemizygous for both driver and effector transgenes (C) were identified by patterns of DsRed2 fluorescence in their eyes and bodies. Wild-type (WT) shows the fluorescence background of wild type non-transgenic *An. stephensi* larvae. Click here for file
