# Supplementary figures and images for: Development of a population suppression strain of the human malaria vector mosquito, Anopheles stephensi
Source: Malar J. 2013 Apr 26;12:142. doi: 10.1186/1475-2875-12-142 (PMC3648444; doi:10.1186/1475-2875-12-142)

## Slide 1
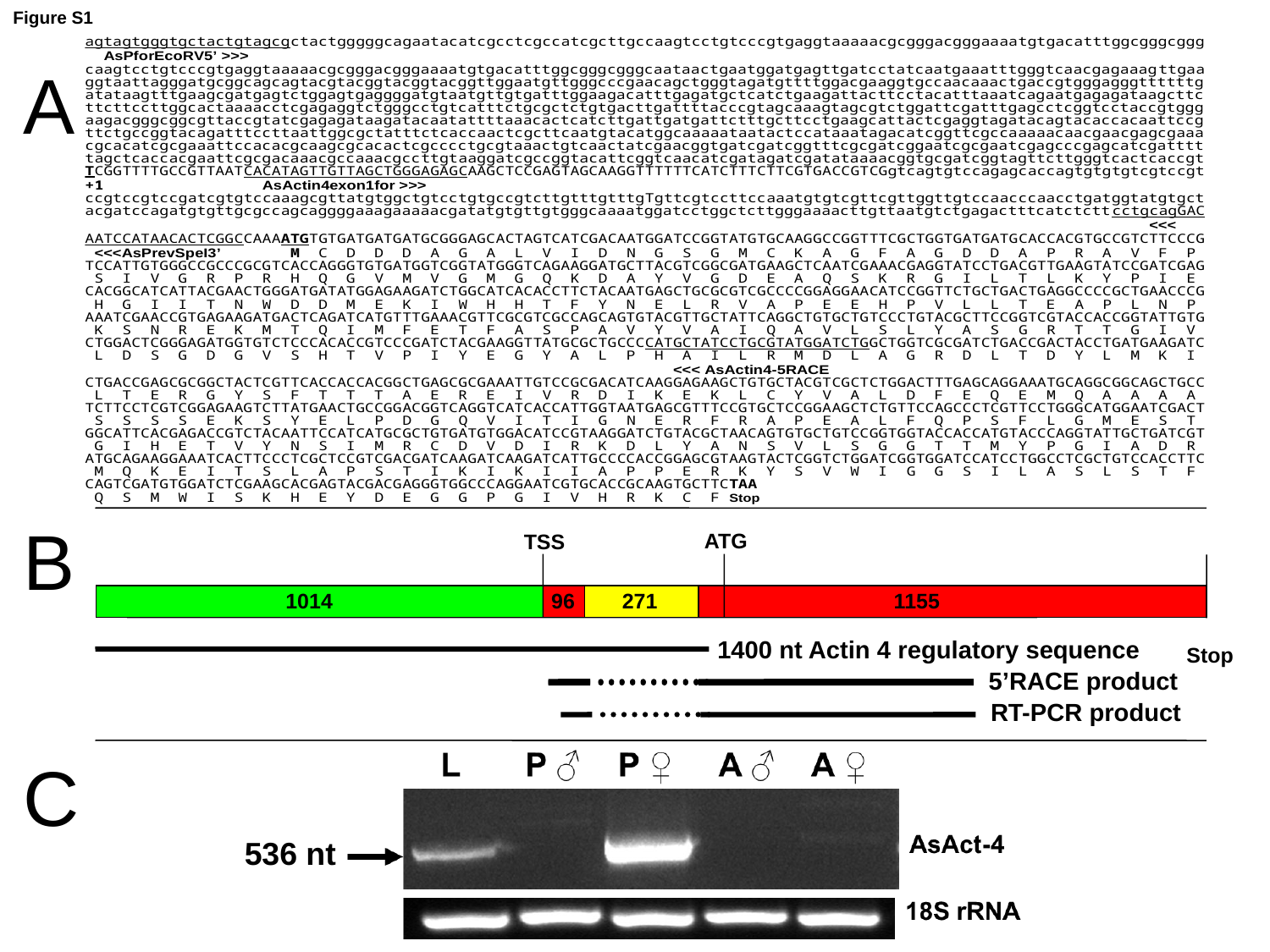

Figure S1
A
B
ATG
TSS
 1014 96 271 1155
 1400 nt Actin 4 regulatory sequence
Stop
 5’RACE product
 RT-PCR product
C
536 nt

Supplement: Additional file 1: Figure S1 — Primary nucleic acid sequence and schematic representation of the Anopheles stephensi Actin-4 gene. A) Capitalized nucleotide sequences correspond to exons 1 and 2, 96 and 1155 nt in length, respectively, while lower case indicates the 1012 nt 5′-end flanking sequence and the 271 nt intron. Underlined sequences indicate the locations of primers described in the manuscript and the transcription (+1) and translation (ATG) start sites are indicated underlined and in bold. B) Exons 1 and 2 (red boxes), the 5′ flanking sequence (green box) and the intron (yellow box) of the An. stephensi Actin-4 gene. Lines underneath indicate the positions of the 1400 nt long AsActin4 regulatory region used to generate AsOX3545, the 5′ RACE product that identified the transcription start-site and the RT-PCR product utilized to determine the gene expression profile. C) Gene amplification analysis of the expression profile of AsAct-4. The 536 nt amplicon is specific for the gene product. Template mRNA samples were derived from larvae (L), pupae (P) and adults (A). An amplification product specific to the 18s rRNA is the positive sample control. [file 1475-2875-12-142-S1.pptx]

## Slide 1
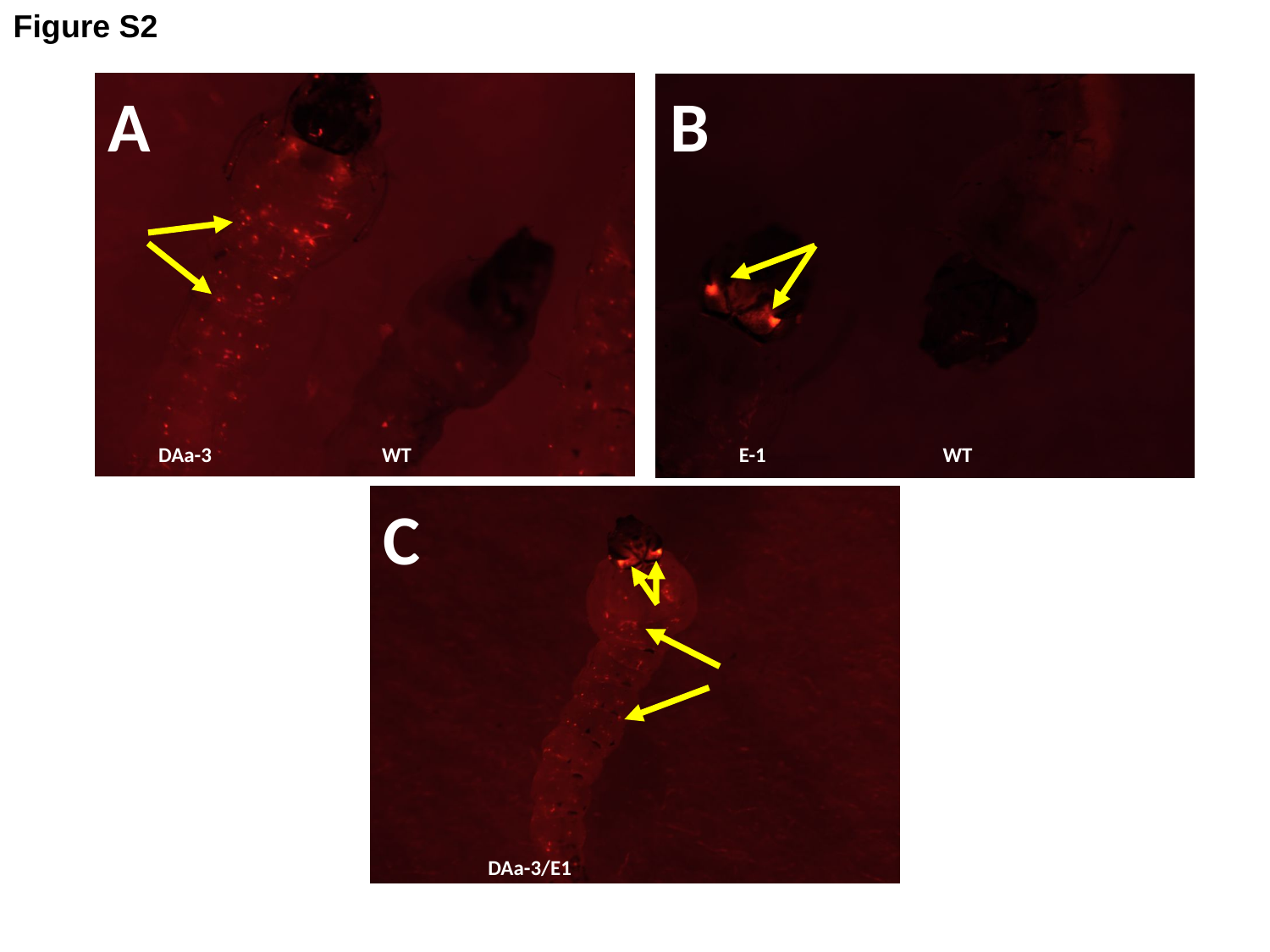

Figure S2
A
B
DAa-3
WT
E-1
WT
C
DAa-3/E1

Supplement: Additional file 3: Figure S2 — Anopheles stephensi larvae hemizygous for driver and/or effector transgenes were identified by patterns of DsRed2 fluorescence consistent with those produced by the 3XP3 and the IE1 promoters. DAa-3 larvae display a scattered fluorescence pattern throughout the body driven by the IE1 promoter (A) while DsRed2 expression in the effector line E-1 is driven by the eye-specific promoter, 3XP3 (B). Larvae hemizygous for both driver and effector transgenes (C) were identified by patterns of DsRed2 fluorescence in their eyes and bodies. Wild-type (WT) shows the fluorescence background of wild type non-transgenic An. stephensi larvae. [file 1475-2875-12-142-S3.pptx]
