# Supplementary material for: Development of a population suppression strain of the human malaria vector mosquito, Anopheles stephensi
Source: Malar J. 2013 Apr 26;12:142. doi: 10.1186/1475-2875-12-142 (PMC3648444; doi:10.1186/1475-2875-12-142)
Supplement: Additional file 2: Table S1 — Sex ratio and survival of Anopheles stephensi driver transgenic lines (DAa-) carrying the construct OX3545. [file 1475-2875-12-142-S2.doc]

**Supplemental materials**

**Table S1. Sex ratio and survival of *Anopheles stephensi*** driver transgenic lines (DAa-) carrying the construct OX3545.

| **G_0_** | **G_1_** | **G_2_** | | | **G_3_** | | **G_4_** | | **G_5_** | |
| --- | --- | --- | --- | --- | --- | --- | --- | --- | --- | --- |
|  | **Transgenic** | **Transgenic** | **Transgenic** | **Transgenic** | **Transgenic** | **Transgenic** | **Transgenic** | **Transgenic** | **Transgenic** | **Transgenic** |
| **adults** | **larvae** | **larvae** | **adults** | **larvae** | **adults** | **larvae** | **adults** | **larvae** | **adults** | **adults** |
| DAa-1 ♂ | 1 ♂ | 325 | 199 | 17 ♂  0 ♀ | 220 | 19 ♂ | 183 | 28 ♂  1 ♀ | 502 | 108 ♂  12 ♀ |
| DAa-2 ♂ | 4 ♂ | 680 | 138 | 22 ♂ 21 ♀ | 134 | 27 ♂  1 ♀ | 120 | 25 ♂  1 ♀ | 141 | 23 ♂  4 ♀ |
| DAa-3 ♂ | 1 ♂ | 200 | 86 | 29 ♂  3 ♀ | 265 | 53 ♂  53 ♀ | 112 | 31 ♂  2 ♀ | 310 | 54 ♂  18 ♀ |
| DAa-4 ♂ | 17 ♂ | 1140 | 659 | 134 ♂ 31 ♀ | 278 | 60 ♂  17 ♀ | 280 | 44 ♂  13 ♀ | 110 | 51♂  14 ♀ |
| DAa-5 ♂ | 4 ♂ | 90 | 45 | 11 ♂ 3♀ | 95 | 19 ♂  9 ♀ | 67 | 14 ♂  4 ♀ | 98 | 28 ♂  4 ♀ |
| DAa-6 ♀ | 1 ♂ | 70 | 44 | 9 ♂  22 ♀ | 40 | 1 ♂  4 ♀ | 119 | 19 ♂  28 ♀ | 90 | 18 ♂  12 ♀ |
| DAa-7 ♀ | 1 ♂ | 100 | 45 | 15 ♂ 9♀ | 88 | 23 ♂  17 ♀ | nd | nd | nd | nd |
| DAa-8 ♀ | 1 ♂ | 90 | 30 | 17 ♂ 10♀ | 89 | 21 ♂  18 ♀ | nd | nd | nd | nd |
| DAa-9 ♂ | 1 ♂  2 ♀ | no progeny | 0 | nd | nd | nd | nd | nd | nd | nd |
| DAa-10 ♂ | 1 ♂ | no progeny | 0 | nd | nd | nd | nd | nd | nd | nd |
| DAa-11 ♂ | 2 ♂ | no progeny | 0 | nd | nd | nd | nd | nd | nd | nd |
| DAa-12 ♂ | 1 ♂ | no progeny | 0 | nd | nd | nd | nd | nd | nd | nd |
| DAa-13 ♂ | 2 ♂ | no progeny | 0 | nd | nd | nd | nd | nd | nd | nd |
| DAa-14 ♂ | 1 ♂ | no progeny | 0 | nd | nd | nd | nd | nd | nd | nd |
| DAa-15 ♂ | 1 ♀ | no progeny | 0 | nd | nd | nd | nd | nd | nd | nd |
